# A Study of Defects in InAs/GaSb Type-II Superlattices Using High-Resolution Reciprocal Space Mapping

**DOI:** 10.3390/ma14174940

**Published:** 2021-08-30

**Authors:** Iwona Sankowska, Agata Jasik, Krzysztof Czuba, Jacek Ratajczak, Paweł Kozłowski, Marek Wzorek

**Affiliations:** Łukasiewicz Research Network—Institute of Microelectronics and Photonics, al. Lotników 32/46, 02-668 Warsaw, Poland; agata.jasik@imif.lukasiewicz.gov.pl (A.J.); krzysztof.czuba@imif.lukasiewicz.gov.pl (K.C.); jacek.ratajczak@imif.lukasiewicz.gov.pl (J.R.); pawel.kozlowski@imif.lukasiewicz.gov.pl (P.K.); marek.wzorek@imif.lukasiewicz.gov.pl (M.W.)

**Keywords:** diffuse scattering, reciprocal space mapping, InAs/GaSb type-II superlattice

## Abstract

In this paper, the study of defects in InAs/GaSb type-II superlattices using high-resolution an x-ray diffraction method as well as scanning (SEM) and transmission (TEM) electron microscopy is presented. The investigated superlattices had 200 (#SL200), 300 (#SL300), and 400 (#SL400) periods and were grown using molecular beam epitaxy. The growth conditions differed only in growth temperature, which was 370 °C for #SL400 and #SL200, and 390 °C for #SL300. A wings-like diffuse scattering was observed in reciprocal space maps of symmetrical (004) GaSb reflection. The micrometer-sized defect conglomerates comprised of stacking faults, and linear dislocations were revealed by the analysis of diffuse scattering intensity in combination with SEM and TEM imaging. The following defect-related parameters were obtained: (1) integrated diffuse scattering intensity of 0.1480 for #SL400, 0.1208 for #SL300, and 0.0882 for #SL200; (2) defect size: (2.5–3) μm × (2.5–3) μm –#SL400 and #SL200, (3.2–3.4) μm × (3.7–3.9) μm –#SL300; (3) defect diameter: ~1.84 μm –#SL400, ~2.45 μm –#SL300 and ~2.01 μm –#SL200; (4) defect density: 1.42 × 10^6^ cm^−2^ –#SL400, 1.01 × 10^6^ cm^−2^ –#SL300, 0.51 × 10^6^ cm^−2^ –#SL200; (5) diameter of stacking faults: 0.14 μm and 0.13 μm for #SL400 and #SL200, 0.30 μm for #SL300.

## 1. Introduction

The presence of defects in semiconductor materials and epitaxial structures has a negative influence on their crystal quality as well as electrical and optical properties. There have been many studies in the last few decades which have focused on the characterization of various defect types [1,2,3,4,5]. In refs. [1,2] dislocations were investigated in detail while in [3,4], more attention was paid to the stacking faults. A much wider classification of defects and their origins was presented in a review article by Mahajan [5]. The author pointed out that threading and misfit dislocations, stacking faults, and twins are typical defects in epitaxial materials. Furthermore, the negative impact of defects on both electrical and optical performance of semiconductor devices was shown by many research groups [6,7]. Thus, to improve device operation and reliability, an effort should be made to grow epitaxial material as close to defect-free as possible. Nowadays, there is a wide range of semiconductor systems used as a basis for various devices. A very interesting one is InAs/GaSb type-II superlattice (SL), which was proposed in 1977 by Sai-Halasz et al. [8]. Since then, considerable progress has been made to use this material in mid-infrared single photodetectors and focal plane arrays. A detailed account of its development history is presented in many review articles, e.g., [9,10,11]. The two greatest difficulties in the epitaxial growth of InAs/GaSb superlattices are the optimization of thermal growth conditions and the precise control of the InSb-like and GaAs-like interfaces (IF), which are the main sources of the strain in the structure. The growth of the superlattice takes place under different thermal conditions (below 400 °C [12]) than those needed for optimal growth of the constituent layers (~430 °C for InAs [13], ~520 °C for GaSb [14]). As a result, the formation of many types of defects (stacking faults, vacancies, interstitial substitutional, and others) can occur [15]. On the other hand, the strain energy is accumulated in InAs/GaSb structure due to the lattice mismatch between interfaces and GaSb substrate. These are +6.3% for InSb-like IF (compressive strain) and −7.2% for GaAs-like one (tensile strain). If the strain becomes too large, the energy can be reduced (strain relaxation) by the generation of misfit dislocations in the structure lattice. This causes a degradation of the crystal quality of the superlattices. However, the strain in the SL can be successfully controlled by the type and the thickness of the interfaces [16,17]. Even for superlattices grown on GaAs substrate, (lattice mismatch between GaSb and GaAs is 7.8%) the dislocation density may be significantly reduced by using either the interfacial misfit array technique [14,18] or metamorphic buffer [19]. Nowadays, the InAs/GaSb superlattices are used more and more in the fabrication of focal plane arrays. In such devices, the performance of every single pixel is crucial for the operation of the entire device. The complex studies on defects reduction in InAs/GaSb superlattices are still carried out by many research groups around the world [20,21]. For example, Walther et al. [22] reported that using a good quality substrate, optimization of the growth, and post-growth processing can result in defects-free single pixels. Despite all the research and technological progress in epitaxial growth, InAs/GaSb superlattices are not yet defect-free. The combined use of several measurement techniques for the identification and characterization of defects is one of the methods leading to the improvement of heterostructure quality by reducing the defect density. Additionally, non-destructive techniques are preferable for these purposes. The high-resolution x-ray diffraction (HRXRD) method is one of them and is successfully used for controlling the quality and structural parameters of InAs/GaSb superlattices, e.g., [16,17,23,24,25,26]. In this case, the HRXRD characterization is usually based on the measurements and analysis of 2θ/ω scans, which do not give full information about the crystal quality of the superlattice. To resolve this issue a detailed analysis of measured reciprocal space maps (RSM) is required, which is an ideal tool for revealing and identification of defects. This is especially the case since different types of defects can result in different shapes of the diffuse scattering. However, for InAs/GaSb superlattices the RSM-based approach is currently only used for the characterization of the lattice relaxation by the generation of misfit dislocations [25,26,27]. To the best of our knowledge, other defects (e.g., stacking faults) have not been investigated so far in this material system in this way.

The main focus of this work is on the identification of defect types (different than misfit dislocations) in InAs/GaSb superlattices by analysis of reciprocal space maps. Three such structures were characterized using the HRXRD technique as well as scanning and transmission electron microscopy. To the best of our knowledge, the distinctive wings-like/streak-like-shaped diffuse scattering in reciprocal space maps, which originates from the stacking faults, has not been presented and analyzed for type-II InAs/GaSb superlattices up till now. Additionally, the influence of the number of periods and growth temperature on the crystal quality of superlattices was examined. The density of the complex defect conglomerates (DC) and the type and size of the defects they are comprised of have been determined.

## 2. Materials and Methods

The superlattices with a nominal period of 9 ML InAs/10 ML GaSb, were grown on GaSb (100) substrates by molecular beam epitaxy in Riber 32P machine using classical effusion cell for In, SUMO type cell for Ga, and valved cracker cells for group V elements (As, Sb). A single period of the superlattice consisted of GaSb and InAs layers and two interfaces. The GaAs-like interface was introduced between GaSb and InAs layers (InAs-on-GaSb interface) using an arsenic soak. For the second one, an antimony soak was employed for the preferential formation of InSb-like bonds (GaSb-on-InAs interface) between InAs and GaSb layers. A lattice match of superlattices to the GaSb substrates was ensured by precise control of both types of interfaces. Superlattices with 400, 300, and 200 periods were grown and labeled as #SL400, #SL300, and #SL200, respectively. The growth rates of binary GaSb and InAs layers (0.5 ML/s) and the beam equivalent pressure for group V/III for GaSb (3.1) were constant for these superlattices. Furthermore, the V/III flux ratio for InAs was set at 4.7 for structures #SL300 and #SL400 and 5.6 for #SL200. The soaking times at GaAs- (t_GaAs_) and InSb-like (t_InSb_) interfaces were 1 s and 3 s for #SL300 and #SL400 superlattices, 2.7 s and 6 s for #SL200, respectively. The growth temperature (T_G_) was 390 °C for #SL300 and 370 °C for #SL400 and #SL200. On the top of #SL300 and #SL400, a 20 nm thick InAs cap layer was grown.

The characterization of the superlattices was carried out using a high-resolution x-ray diffractometer of PANalytical X’Pert PRO. Detailed configuration of the setup is given in [28]. Both 2θ/ω curves and reciprocal space maps were measured. The basic information, such as the thickness of the period (d_SL_), thicknesses of layers in the period, and values of perpendicular lattice mismatch (Δa/a) about investigated superlattices were obtained from the analysis of diffraction curves. The crystal quality of the structures was also investigated using high-resolution transmission electron microscopy (HRTEM). The examination of defects was based on the analysis of reciprocal space maps measured around 004 GaSb reciprocal lattice point and images from a scanning electron microscope (SEM). To confirm the types of the defects, plan-view images were made using a transmission electron microscope (TEM). In addition, the results of the reciprocal space mapping investigation of dry-etched #SL400 superlattice are presented in a later section. The sample was etched using Inductively Coupled Plasma (ICP)—Reactive Ion Etching (RIE) in ICP-RIE Oxford Plasmalab 100 system. Every process was conducted in 1:4 ratio BCl_3_:Ar plasma at a pressure of 4 mTorr. Both ICP and RF power was set at 100 W. This resulted in the etch rate of about 72.5 nm/min. Three consecutive selective etchings were done. One hundred periods were removed during each etching. The thickness of the structure after each step was controlled using KLA-Tencor P-16 contact profiler.

## 3. Results and Discussion

**Figure 1:**
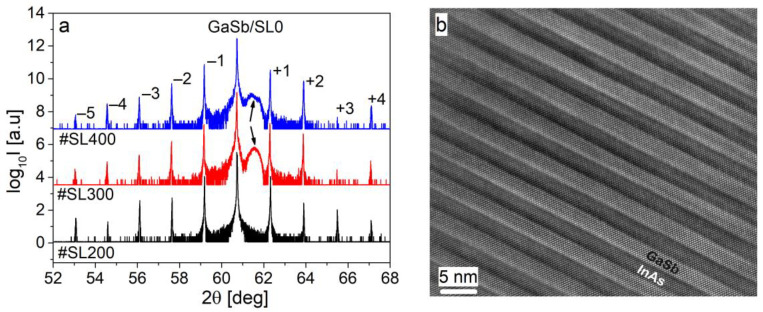
(**a**) Measured 2θ/ω curves for superlattices #SL400, #SL300, and #SL200. Peaks from thin InAs cap layers present in #SL300 and #SL400 are marked by arrows. (**b**) HRTEM image of #SL200 superlattice—the successive InAs and GaSb layers are visible.

High intensity and narrow superlattice peaks up to −5th and +4th orders were observed on HRXRD curves measured for #SL400, #SL300, and #SL200 structures (Figure 1a). This indicates a high crystal quality of the superlattices and a constant period thickness during the growth process. Wide peaks (marked by arrows) visible in curves for #SL300 and #SL400 originate from the InAs cap layer. The crystal quality of the superlattices was also confirmed using cross-sectional HRTEM images. The well-defined InAs and GaSb layers can be observed for all investigated samples. An exemplary image of the #SL200 structure is shown in Figure 1b.

Values of perpendicular lattice mismatch measured between the zeroth order superlattice peak (SL0) and GaSb substrate are 0 ppm for #SL300, −180 ppm for #SL400, and −360 ppm for #SL200. The analysis of 2θ/ω curves confirmed that 9 ML of InAs and 10 ML GaSb thick layers were grown. Furthermore, the thicknesses of GaAs-like IF and InSb-like IF were determined from simulation to be 0.6 ML and 1.54 ML, respectively. In simulations, a binary InSb layer was assumed as InSb-like IF. A ternary GaAs_x_Sb_(1−x)_ layer was simulated as a GaAs-like interface, where x values were 0.88, 0.925 and 0.96 for #SL300, #SL400 and #SL200, respectively. As can be seen, the T_G_ difference of 20 °C for #SL300 and #SL400 (all other growth parameters were the same) caused a change of the chemical composition of GaAs-like interface, which resulted in Δa/a value of −180 ppm. A twice longer t_InSb_ and t_GaAs_ for #SL200 caused an increase of InSb IF thickness and a larger value of x in ternary GaAs_x_Sb_(1−x)_ IF compared to #SL400, which was grown at the same T_G_ as #SL200. As a result, perpendicular lattice mismatch was further increased to −360 ppm. It can be concluded that the GaAs-like IF had a greater impact on the change of Δa/a than InSb-like IF. These differences between the superlattices caused very little shift in the angular positions of their satellite peaks (Figure 1a). This means that the thicknesses of the periods were comparable. The following values of d_SL_ were obtained from the simulations of 004 diffraction curves: #SL400—65.3 Å, #SL300—65.4 Å, and #SL200—65.5 Å.

**Figure 2:**
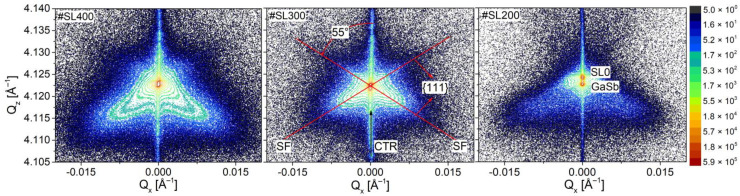
Distribution of diffuse scattering in reciprocal space maps measured along [110] direction for 004 reflection for #SL400 (400 periods), #SL300 (300 periods) and #SL200 (200 periods).

In Figure 2, reciprocal space maps for three superlattices measured around the GaSb 004 reciprocal lattice point are shown. To provide comparability between the structures the same measurements settings for each RSM were used. The reciprocal lattice points of GaSb substrate and SL0 are close to each other for samples #SL400 and #SL200 and overlap in the case of #SL300, due to a small lattice mismatch. They are narrow for all studied superlattices. Furthermore, the crystal truncation rod (CTR) along the specular reflection can be observed as well. Diffuse scattering (DS) around reciprocal lattice points was also detected for all characterized structures.

The DS has a wings-like regular shape and its intensity is focused along the 55° angle with respect to the [001] direction. This corresponds very well with the value of 54.7°, which is typical for the stacking faults (SF)—planar defects localized in {111} planes [29]. The intensity of streak-like stacking faults is mostly focused at lower values of Q_z_ with respect to GaSb reciprocal lattice point. According to e.g., [30,31,32], different types of defects can cause a shift of diffuse scattering intensity along the Q_z_ axis: interstitial defects—to positive values and vacancies—to negative values of Q_z_. Therefore, it can be concluded that apart from stacking faults, there are also vacancies present in the investigated #SL200, #SL300, and #SL400 superlattices. Different intensity distributions of DS caused by stacking faults were observed for investigated superlattices. For #SL300 DS is mostly focused around 004 reciprocal lattice point in both Q_x_ and Q_z_ directions. For #SL400 and #SL200, the wings of DS are more elongated in the direction of smaller Q_z_ values, and the widening in the Q_x_ is also more pronounced than for #SL300. The analysis of defects, which are responsible for different shapes and distributions of DS is presented in the following subsections.

### 3.1. Simple Defect Analysis

**Figure 3:**
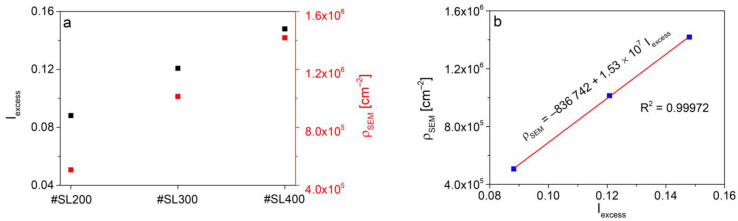
(**a**) The excess intensity (black points) obtained from the integration of diffuse scattering and defects density (ρ_SEM_) (red points) estimated from SEM images made for investigated superlattices. (**b**) Linear dependence of ρ_SEM_ as a function of excess intensity (blue points—experimental data, red line—linear fit).

In this approach, the integration of diffuse scattering in reciprocal space was performed. It is known that the defects present in the crystals are sources of diffuse scattering. Wang and Matyi showed that the integrated diffuse intensity (I_excess_) can be a good measure of crystal damage [33]. This method was previously used by us to determine the dislocation density in quantum cascade laser structures [28]. The entire integration procedure was thoroughly described in that paper. As a reminder, the I_excess_ can be defined as:

(1) $$I_{\mathrm{excess}}=\int_{Q_{z,\min}}^{Q_{z,\max}}\int_{Q_{x,\min}}^{q_{\mathrm{xa}}}I_{\mathrm{net}}\left(Q_{x},Q_{z}\right){\mathrm{dQ}}_{x}{\mathrm{dQ}}_{z}+\int_{Q_{z,\min}}^{Q_{z,\max}}\int_{q_{\mathrm{xb}}}^{Q_{x,\max}}I_{\mathrm{net}}\left(Q_{x},Q_{z}\right){\mathrm{dQ}}_{x}{\mathrm{dQ}}_{z}$$

where Q_x,min_ = −0.02 Å^−1^, Q*_x_*_,max_ = 0.02 Å^−1^, Q_z*,*min_ = 4.105 Å^−1^ and Q*_z_*_,max_ = 4.140 Å^−1^ are the limits of symmetrical reciprocal space maps shown in Figure 2; q_xa_ and q_xb_ are the limits which assure that the coherent scattering intensity is not taken into account during the integration procedure. These limits were defined based on the analysis of RSM for a high-quality superlattice (reference sample #SL with 300 periods) following [28]. The excess intensity values of 0.0882; 0.1208 and 0.1480 were obtained for #SL200, #SL300 and #SL400, respectively. The largest integrated intensity of diffuse scattering was obtained for the superlattice with 400 periods, and the smallest for the superlattice with the 200 periods (black points in Figure 3a).

**Figure 4:**
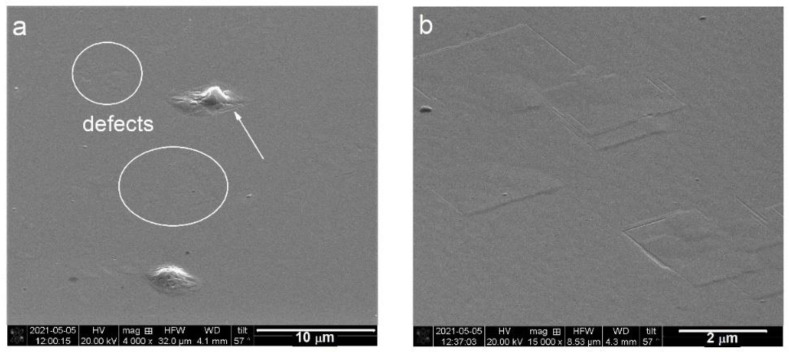
(**a**) SEM image of rectangular-shaped flat defects without (in circles) and with (marked by arrow) precipitates in the center for #SL400; (**b**) A higher magnification SEM image of flat defects.

The SEM characterization was also carried out within the scope of the *simple defect analysis*. Obtained images revealed a few by few micrometers in size, rectangular-shaped defects on the surfaces of investigated superlattices. Examples of these, found on the surface of #SL400 are shown in Figure 4. The majority of the defects were flat (in circles in Figure 4a) and some of them had precipitates in their centers (marked by an arrow in Figure 4a). The former are shown under higher magnification in Figure 4b. The defect size estimated from SEM images is (2.5–3) μm × (2.5–3) μm for #SL200 and #SL400, and (3.2–3.4) μm × (3.7–3.9) μm for #SL300.

The defect density (ρ_SEM_) was calculated by counting all flat defects and dividing their number by area corresponding to the SEM image field of view shown in Figure 4a. The tilt angle was taken into account. Calculated ρ_SEM_ values of 1.42 × 10^6^ cm^−2^ for #SL400, 1.01 × 10^6^ cm^−2^ for #SL300, and 0.51 × 10^6^ cm^−2^ for #SL200 are presented in Figure 3a (red points). The same as for I_excess_(number of periods) function, the value of defect density increases with the number of periods in the superlattice. Additionally, a linear dependence between ρ_SEM_ and integrated intensity was observed (Figure 3b). Even though the #SL300 was grown at a higher temperature than other samples, no correlation between the number of defects and the growth temperature of the superlattice was observed at this stage.

### 3.2. Detailed Defect Analysis

**Figure 5:**
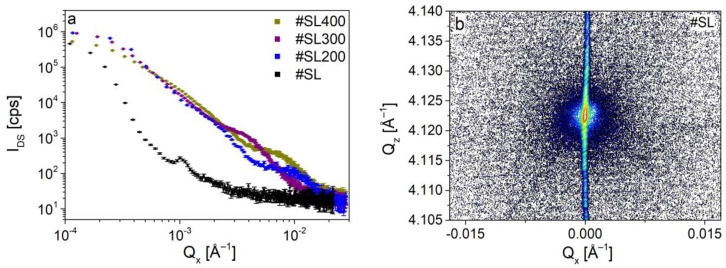
(**a**) The distribution of diffuse scattering intensity as a function of Q_x_ for investigated samples #SL400, #SL300, #SL200. Black points were obtained for reference sample #SL. (**b**) The reciprocal space map measured around 004 GaSb reflection for the reference sample (#SL).

In this subsection, a detailed investigation of diffuse scattering intensity (I_DS_) by analyzing the log-log plot of I_DS_ (Q_x_) is presented. For this purpose, the cross-sections of DS surfaces along Q_z_ for different values of Q_x_ were created for investigated superlattices. Obtained curves including one for the reference sample are shown in Figure 5a.

A rapid decrease of I_DS_ in the Q_x_ range of 1 × 10^−4^ Å^−1^–2 × 10^−3^ Å^−1^ for the reference sample was observed. A low intensity of diffuse scattering is visible for #SL (Figure 5b), however, it does not degrade its high crystal quality. For values of Q_x_ > 2 × 10^−3^ Å^−1^ the log(I_DS_) = *f*(log(Q_x_)) function is constant and fixed at the background level. The peak at 1 × 10^−3^ Å^−1^ corresponds to the monochromator streak, which is well detected for high crystal quality structures. The curves for #SL400, #SL300, and #SL200 can be divided into distinctive segments following the formula I_DS_~Q_x_*^−m^*. The index *m* indicates a specific type of diffuse scattering, namely Huang, Stokes-Wilson, or thermal, which are closely related to the Q_x_ values. The experimental points for small Q_x_ up to ~3.5 × 10^−4^ Å^−1^ showed linear dependence in log-log scale with *m* close to unity for all characterized superlattices. This region can be associated with Huang scattering, which dominates close to Bragg reciprocal lattice points, far from the defects. In the adjacent region, for Q_x_ up to ~2.21 × 10^−3^ Å^−1^, ~3.70 × 10^−3^ Å^−1^, and ~5.32 × 10^−3^ Å^−1^ for #SL300, #SL400, and #SL200, respectively, I_DS_ decreases with *m* being equal to ~3. This behavior is due to the Stokes-Wilson diffuse scattering, which originates from the vicinity of the defects. As was reported in [34] the radius of the defects can be calculated from the following relation:

(2) $$R=\frac{\pi }{Q_{x0}},$$

where Q_x0_ is the value, at which *m* changes from 1 to 3 for Q_x_^−m^ relation. The following diameters of defects (2R) were obtained for characterized structures: ~2.45 μm (#SL300), ~2.01 μm (#SL200), and ~1.84 μm (#SL400).

**Figure 6:**
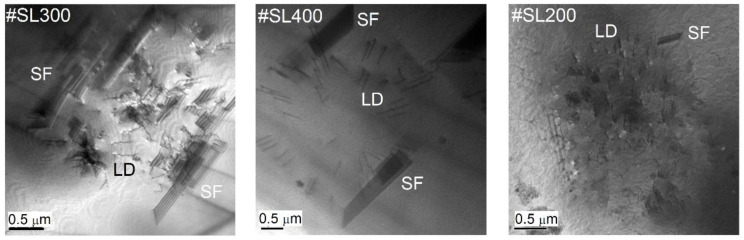
The TEM plan-view images of defect conglomerates consisting of stacking faults (SF) and linear dislocations (LD) for the #SL300, #SL400, and #SL200 structures.

For larger values of Q_x_, a quick decrease of I_DS_ should occur due to thermal diffuse scattering. Instead, I_DS_ again follows ~Q_x_^−1^ relation for of Q_x_ in the range of (~2.21 × 10^−3^ Å^−1^–~3.89 × 10^−3^ Å^−1^) for #SL300, (~3.70 × 10^−3^ Å^−1^–~7.80 × 10^−3^ Å^−1^) for #SL400, and (~5.32 × 10^−3^ Å^−1^–~1.09 × 10^−2^ Å^−1^) for #SL200. Furthermore, this behavior repeats itself as I_DS_ decreases with *m*~3 and then again *m*~1. Such occurrence indicates the presence of additional smaller defects in these structures. The TEM plan-view images were taken to confirm this supposition and to identify the types of these defects. The images revealed that in all characterized superlattices the defect conglomerates were present. In Figure 6, examples of these are shown for each sample.

The distance between the conglomerates is in the range from several to tens of micrometers for #SL400 and #SL300, and from tens to hundreds of micrometers for #SL200. A high DC density was observed for samples #SL400 and #SL300. The largest number of defect conglomerates was observed for #SL400, while the smallest was for #SL200. This is in good agreement with the results obtained from *simple defect analysis*, where the values of both I_excess_ and ρ_SEM_ increase with the number of periods in superlattices. Furthermore, the size of DC of about three micrometers corresponds very well with the size of the flat defects. Therefore, it can be concluded that flat defects in SEM images are the defect conglomerates identified during TEM plan-view characterization. The presented analysis of diffuse scattering intensity distribution as a function of Q_x_ also indicates the presence of micrometer-sized defects in characterized superlattices. Based on TEM images, these were composed of both stacking faults and linear dislocations (LD). Moreover, it was noticed that the number of stacking faults in the defect conglomerates differed significantly between the structures. The largest SF number was observed for the #SL300 and the smallest for #SL200. The intensity of diffuse scattering in Figure 5 in the range of (~2.21 × 10^−3^ Å^−1^–~3.89 × 10^−3^ Å^−1^) for #SL300, (~3.70 × 10^−3^ Å^−1^–~7.80 × 10^−3^ Å^−1^) for #SL400, and (~5.32 × 10^−3^ Å^−1^–~1.09 × 10^−2^ Å^−1^) for #SL200 showed the same trend, i.e., the largest values for structure #SL300 and the smallest for #SL200. Average intensities (I_SFavg_) in these regions were 1650 cps, 450 cps, and 130 cps for #SL300, #SL400, and #SL200, respectively.

To determine the stacking fault diameter, we used streak-like diffuse scattering associated with the presence of these defects in investigated structures and followed the reports in [35]. Among other things, the authors showed that the full width at half maximum (FWHM) of the stacking-faults-related streak was practically constant along the [111] direction. Furthermore, the following equation can be used to calculate the diameter (d_SF_) of SF:

(3) $$d_{\mathrm{SF}}=\frac{2\pi }{\mathrm{FWHM}}$$

**Figure 7:**
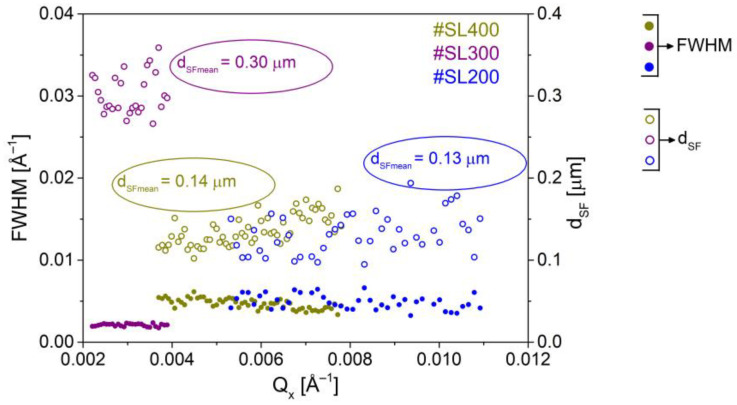
The full width at half maximum (FWHM) of diffuse scattering streak from the stacking faults (full circles, left vertical axis); Diameters of stacking faults calculated using FWHM (open circles, right vertical axis).

The widths of stacking faults along the streak for studied superlattices are nearly constant, (full circles in Figure 7). The SF diameters were calculated using (3) (open circles in Figure 7), after which the average values for each sample were determined. The largest stacking faults with the average size of 0.30 μm were present in #SL300, while twice smaller of 0.14 μm and 0.13 μm were in #SL400 and #SL200, respectively.

The results of the *detailed defect analysis* confirmed the existence of micrometer-sized defects in characterized superlattices as was shown in the *simple defect analysis*. Additionally, the presence of smaller defects (stacking faults) in these structures was revealed based on the behavior of the log(I_DS_) = *f*(log(Q_x_)) function and confirmed by TEM plan-view characterization. The latter showed that flat defects observed on SEM images were composed of smaller ones—stacking faults and linear dislocations.

The summary of defect-related parameters obtained from *simple* and *detailed defect analyses* is presented in Table 1.

These two methods complement each other in the following way. The first one provided information about the degree of structural damage (I_excess_ and ρ_SEM_) and the size of defect conglomerates. The latter was further confirmed in the *detailed defect analysis* (DC size and 2R), which also revealed the presence of smaller defects.

### 3.3. Analysis of the In-Depth Distribution of Defects

**Figure 8:**
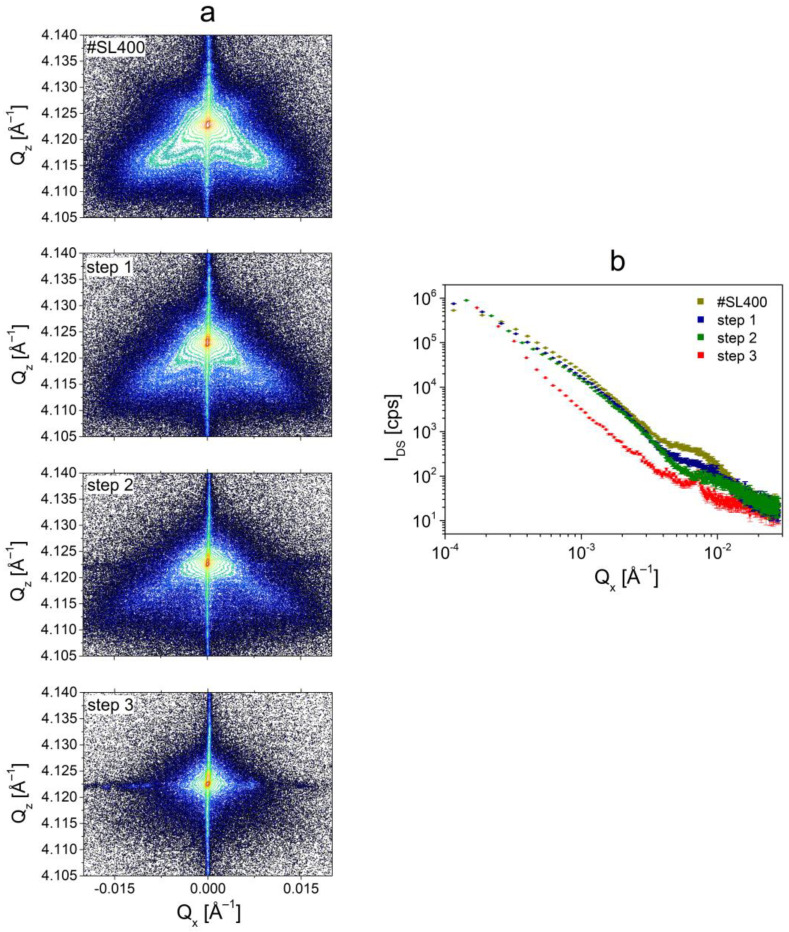
(**a**) Symmetrical reciprocal space maps of #SL400 superlattice before and after each step of dry etching. (**b**) The distribution of I_DS_ as a function of Q_x_ for #SL400 before and after each etching.

In this subsection, the in-depth distribution of defects within the superlattice was investigated. For this purpose, the #SL400 structure was subjected to a cycled dry etching—HRXRD measurement process. Three etchings were performed, of which each removed one hundred periods. As a result, the superlattices with 300 (step 1), 200 (step 2), and 100 (step 3) periods were obtained. The reciprocal space maps presented in Figure 8a show the decrease of streak-like diffuse scattering after each etching. For superlattice with 300 periods (step 1), the smear of stacking faults (wings) comparable to the source structure (#SL400) was observed. After the second etching (step 2) the shape of the DS was similar to that observed for #SL200. The diffuse scattering originating from stacking faults vanished after the last etching (step 3).

The methodology described in the previous subsection was also employed in this case. The decrease of diffuse scattering intensity in the range of Q_x_ corresponding to the stacking faults for structures marked as step 1 and step 2 was observed (Figure 8b). Furthermore, for the step 3 designated sample the I_DS_ associated with stacking faults vanished. It was also observed that the diffuse scattering intensity for ~3.7 × 10^−4^ Å^−1^ < Q_x_ < ~3.7 × 10^−3^ Å^−1^ was about 3–5 times smaller than for #SL400, step 1, and step 2 samples. For these three, a small decrease of intensity occurred with each etching in the aforementioned Q_x_ range.

The circular residual diffuse scattering visible for the step 3 sample was probably related to dislocations that were revealed in TEM plan-view images (Figure 6), which could still be present in the structure. A similar shape of diffuse scattering from different types of dislocations was shown in [29]. However, the effects related to the damage of the surface during the etching were also visible in the reciprocal space map for the step 3 sample (the horizontal line of raised background). This effect was observed in the form of a peak at Q_x_ ~ 0.0070 Å^−1^ in Figure 8b.

**Figure 9:**
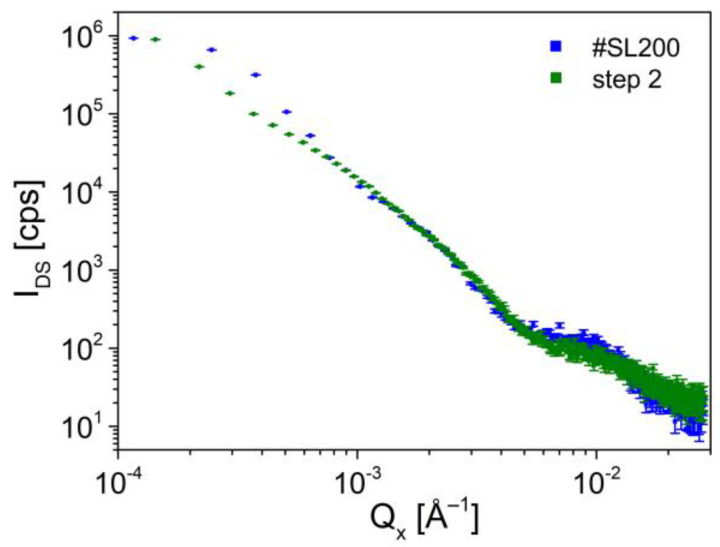
The comparison of log(I_DS_) vs. log(Q_x_) distributions for #SL200 and step 2 samples.

In Section 3.1, it was shown that there is a linear dependence between the values of defect density calculated from SEM images and the integrated intensity for samples with a different number of periods, (Figure 3b). To investigate the defect density vs. SL thickness, the integration of diffuse scattering intensity for step 1 and step 2 samples was performed. The density values were calculated using the equation given in Figure 3b. Sample step 3 was excluded from the analysis due to significant surface damage, which could affect the result of the integration. As can be seen in Figure 8a, this effect is also visible after the second etching (the horizontal line of raised background). However, the diffuse scattering related to defects is dominant and the contribution from surface damage can be neglected. This assumption was confirmed by comparing the log(I_DS_) vs. log(Q_x_) for #SL200 and step 2 sample with 200 periods left (Figure 9). The intensities in the ranges corresponding to both larger defects and stacking faults were almost the same for these two samples.

The following values of I_excess_ were obtained from the integration of measured diffuse scattering for etched #SL400 structure: 0.1139 for step 1 and 0.0828 for step 2. Using these values of I_excess_, the defect densities after each etching were calculated and they were equal to 0.91 × 10^6^ cm^−2^ and 0.43 × 10^6^ cm^−2^ for step 1 and step 2, respectively. These values are in very good agreement with ρ_SEM_ obtained from SEM analysis (1.01 × 10^6^ cm^−2^ for #SL300 and 0.51 × 10^6^ cm^−2^ for #SL200). Small differences can be caused by a difficulty in the precise control of the etching depth, which could result in the exact number of periods removed during each etching being either less or more than one hundred. Taking this into account we believe that the defect conglomerates are uniformly distributed in SL volume as every 100 periods increases the sheet DC density by about 0.48–0.51 × 10^6^ cm^−2^. Additionally, based on the comparison of I_DS_ distributions shown in Figure 9, a similar relation was observed for stacking faults in samples grown at the same temperature. These results are useful for further optimization of SL growth aimed at the reduction of SF density. The minimum and sufficient thickness of SL is 200 periods. In the RSM measured for 100 periods, there is no streak-like diffuse scattering, which makes it impossible to analyze the defects. In turn, each subsequent SL period over the number of 200 does not provide any additional information about defects and is therefore redundant.

## 4. Conclusions

The crystal quality characterization of three #SL400, #SL300, and #SL200 type-II InAs/GaSb superlattices was described. The diffuse scattering was measured by reciprocal space mapping. Its wings-like shape indicated the defect type to be stacking faults.

S*imple and detailed defect analyses* were proposed. The former revealed the existence of micrometer-sized defects. It was also shown that the values of integrated reciprocal space intensity and defect density calculated from SEM images increased with the number of periods in superlattices, regardless of the growth temperature. The *detailed defect analysis* provided information about the types of defects and the detailed distribution of their intensity. The presence of micrometer-sized defect conglomerates was also confirmed using TEM plan view images. It was shown that the conglomerates were comprised of stacking faults and linear dislocations. The number of stacking faults was the largest for the #SL300 superlattice, which was grown at a higher temperature than the other two. The analysis of the distribution of diffuse scattering intensity as a function of Q_x_ confirmed this. The average value of diffuse scattering intensity from stacking faults for the #SL300 was about four and thirteen times higher than the intensity for #SL400 and #SL200, respectively. The sizes of the stacking faults were calculated from the FWHM of the diffuse scattering streak. Stacking faults of the largest size were observed for the superlattice (#SL300) grown at the highest temperature (390 °C). The structures grown at the same temperature (370 °C) had stacking faults of similar sizes, regardless of the number of periods (#SL200 and #SL400). The number of micrometer-sized defects increased with the increasing thickness of the structure. On the other hand, the number of stacking faults depended on the growth temperature of the structure: the higher the temperature, the more stacking faults. Moreover, the SF diameter is directly proportional to the growth temperature, however, it is not affected by the number of periods.

The analysis of the in-depth distribution of defects in the #SL400 superlattice showed that their number decreased with each etching. After the removal of 300 periods, the wings-like diffuse scattering vanished due to the negligibly low density of stacking faults in 100 periods left. Therefore, the optimal number of periods for further optimization of SL growth aimed to reduce the SF density is 200. 

## Figures and Tables

**Table 1:** The summary of *simple* and *detailed defect analysis* results. The values of I_excess_, 2R, d_SFmean_, and I_SFavg_ were obtained using the HRXRD technique, the values of DC size and ρ_SEM_ from SEM and TEM characterization.

Sample	I_excess_	DC Size [μm × μm]	ρ_SEM_× 10^6^ [cm^−2^]	2R [μm]	d_SFmean_ [μm]	I_SFavg_ [cps]
***simple defect analysis***						
#SL400	0.1480	(2.5–3) × (2.5–3)	1.42	-	-	-
#SL300	0.1208	(3.2–3.4) × (3.7–3.9)	1.01	-	-	-
#SL200	0.0882	(2.5–3) × (2.5–3)	0.51	-	-	-
***detailed defect analysis***						
#SL400	-	~3 × ~3	-	~1.84	0.14	450
#SL300	-	~3 × ~3	-	~2.45	0.30	1650
#SL200	-	~3 × ~3	-	~2.01	0.13	130

## Data Availability

The data is available from the corresponding author upon reasonable request.
